# Precision medicine in Australia: now is the time to get it right

**DOI:** 10.5694/mja2.51777

**Published:** 2022-11-27

**Authors:** Rosie O'Shea, Alan S Ma, Robyn V Jamieson, Nicole M Rankin

**Affiliations:** ^1^ University of Sydney Sydney NSW; ^2^ Western Sydney Genetics Program Children's Hospital at Westmead, Sydney Children's Hospitals Network Sydney NSW; ^3^ Centre for Health Policy University of Melbourne Melbourne VIC

**Keywords:** Health services research, Genetic testing, Molecular medicine

Implementation science based health care research is urgently needed for genomic and precision medicine in Australia

Precision medicine is a tailored approach to health, incorporating an individual's genetic make‐up, environment and lifestyle, and is a new frontier offering much promise for disease prevention and cure.^1^ Its recent rise has been largely driven by rapid advances in genomic medicine, with sequencing of an individual's genetic code identifying opportunities for precision health care, therapies and diagnostics. Genomics has revolutionised many areas, including public health (eg, population genetic carrier screening and pathogen genomic sequencing during the coronavirus disease 2019 [COVID‐19] pandemic), pharmacogenomics (drug metabolism and response genes), cancer management (tumour sequencing for diagnosis and therapy targets), pregnancy management (testing, screening and pre‐implantation genetic diagnosis) and rare diseases (Box 1). Medicare item numbers are now approved for genomic diagnostics in cancer, pre‐implantation genetic diagnosis, and certain paediatric, renal and cardiac conditions. New genetic therapies have arisen, including Therapeutic Goods Administration (TGA)‐funded ocular (voretigene neparvovec; Luxturna, Novartis), neuromuscular (onasemnogene abeparvovec; Zolgensma, Novartis), oncological (tisagenlecleucel; Kymriah, Novartis) and other genetic therapies.

Box 1 **Opportunities, benefits, barriers and examples in the implementation of precision medicine into health care**

**Table:** 

**Precision medicine facilitators**	**Benefits and role in precision medicine**	**Barriers to adoption into standard health care**	**Implementation science approaches to address barriers**
Medicare funding for genomic tests in medicine (eg, clinical paediatrics, renal, cardiac, and other medical specialty areas) as well as state‐based funding for other conditions	Improve time to diagnosis and management and avoid lengthy expensive diagnostic odyssey Personalised reproductive counselling and accurate recurrence risk information including uptake of pre‐implantation genetic diagnosis/in vitro fertilisation Unlocks NDIS funding and future precision therapies when available	**Provider:** lack of education in genomics and time to use by non‐genetics professionals **Organisational:** few strategies to ensure equity of access for CALD*, Aboriginal and Torres Strait Islander, and rural and remote patients **Health system/policy:** inequity due to different state‐based funding models for genomic testing and Medicare accessibility	**Pre‐implementation studies** to address systemic barriers before releasing Medicare items, involving key local, health care, and systems/policy stakeholders, including close engagement with Aboriginal and Torres Strait Islander communities, and CALD* consumers and codesign of implementation strategies **Frameworks:** the use of the Genomic Medicine Integrative Research (GMIR) framework^2^ would allow for health care planning and service delivery approaches that are feasible and sustainable Genomic education and multidisciplinary practices are strategies used to deal with the issue of genomic mainstreaming in medicine^3^
Genomic oncology initiatives such as Medicare funding, germline and somatic testing strategies to inform therapy, novel targets and family risk	Tailor management and prognosis/monitoring (eg, PARP inhibitors and *BRCA*) Allow asymptomatic family testing and proactive screening Increasingly used for MTB and targeted therapies for increased cancer survival	**Provider:** need for role delineation issues, as surgeons/oncologists are not familiar with using genomics in daily practice **Organisational:** lack of genomic education and support for non‐genetic oncologists and workforce shortages affecting MTB and genetic counselling services **Health system:** limited funding and infrastructure for MTB approach	**Frameworks** such as the Consolidated Framework for Implementation Research (CFIR) can be used to both identify gaps and strategies that can address the barriers Adopting electronic medical record systems, interdisciplinary collaborations between genetic counsellors and oncology, identifying genomic champions and adaptable learning health systems have been identified as strategies for implementation in oncology^3^
Advanced therapeutics such as cell and gene therapies and clinical trials (ocular; neurological, such as SMA; cancer, such as CAR‐T; and many more in future)	Vision restoration for inherited blindness, survival for previously incurable diseases (SMA), and curative/treatment options for difficult cancers (CAR‐T)	**Provider:** limited expertise in administration **Organisational:** lack of equitable pathways to access a genetic diagnosis and advanced therapy **Health system:** high cost of therapies **Policy:** ethical choices for high cost therapies, clinical trial access, and taxpayer burden	**Process models** such as the knowledge‐to‐action cycle^4^ can guide the stages of research from pre‐implementation, identifying local contextual implementation factors, assessing the barriers and facilitators, and selecting and tailoring strategies, such as new models of care, that are codesigned to address the diagnostic and access pathways, equity, feasibility, sustainability and acceptability of advanced therapeutics in existing health care systems
Population screening (eg, reproductive health with non‐invasive prenatal screening, pre‐conception carrier screening, pre‐implantation genetic diagnostics and newborn genomic screening) and public health sequencing (eg, pathogen genomics)	Population screening for large number of rare but severe genetic conditions including cancer, pregnancy planning options for couples at risk, and non‐invasive pregnancy screening options, and possible future genomic screening for a wide range of diseases and therapeutic targets Pathogen genomics complements rapid diagnosis, epidemiological surveillance and management of pandemic response (eg, COVID‐19)	**Provider:** lack of genomic education for primary care and reproductive health practitioners **Health systems:** lack of pathways to implement broad‐based public health screening at a population level, including consent and counselling **Policy:** societal and ethical issues, high cost of huge number of genomic tests in population screening	**Evaluation frameworks** such as RE‐AIM (Reach, Effectiveness, Adoption, Implementation and Maintenance) plan and can be used to assess barriers, plan research, and evaluate outcomes This strategy was used to evaluate two programs of population DNA screening in the United States, showing evidence of gaps and implementation challenges for future screening programs^5^
Future precision health technologies (eg, pharmacogenomics, polygenic risk profiles, direct to consumer testing, and predictive genomic testing informing lifestyle)	Individualised/personalised medicine based on genomic risk profiles could affect medication advice/dosage, lifestyle, and a wide range of health implications	**Provider:** lack of trained health care professionals to cope with influx and demand, and potential exploitation of patients by private providers **Policy and health systems:** lack of evidence base for these frontier technologies, models of care, and lack of public genomic literacy	**Pragmatic, real‐world trials** of implementation models for precision medicine are required to demonstrate scalability and adaptability to new areas of medicine This approach has been trialled with pharmacogenomics in primary care^6^

CALD = culturally and linguistically diverse; CAR‐T = chimeric antigen receptor T cells; COVID‐19 = coronavirus disease 2019; MTB = multidisciplinary tumour boards; NDIS = National Disability Insurance Scheme; SMA = spinal muscular atrophy. Even though many new advances and opportunities exist in genomics and precision medicine, unlocking these benefits and overcoming potential barriers is a significant issue. Implementation science based research approaches^7^, ^8^, ^9^ are required at a local, health care and systemic level to select the best strategies to ensure that tailored interventions will overcome contextual barriers for each target environment, promoting the adoption of new practices into standard care.

It is an exciting time for genomic and precision medicine in Australia. An ever‐increasing proportion of families are receiving accurate genetic diagnoses, access to screening and counselling, and clinical management from publicly funded genomic technologies (Box 1), with other areas under investigation and a focus towards future government funding.^10^, ^11^ However, despite the clinical benefits of genomics, the uptake in the clinic and bedside for patient care to access publicly funded new diagnostics and therapies is far from equitable or routine in Australia.^12^ Many challenges and barriers are known, with others yet to be documented (Box 1). At the clinician level, many non‐genetics professionals are not well prepared to use the newly funded genomic diagnostic tests. Medical and training curricula covering genetics and genomics require updating, including guidance from professional bodies and colleges, both in primary care and specialty groups such as ophthalmology.^13^, ^14^ Even though many clinicians report they would rather refer to local genetics services or professionals to perform genomic testing, interpretation and clinical management of cases, the current genetics workforce in Australia is inadequate, with only an estimated 150 genetic physicians and 220 genetic counsellors in the country.^15^ Many clinical service waitlists have expanded to years rather than months.^16^ This reflects a significant worldwide issue, with up to 44% shortfall in the genetics workforce.^17^ At an organisation level, health care systems are struggling to adopt new genomic innovations, even when there is proven validity and utility.^18^

The translation gap between medical evidence‐based practices and actual clinical adoption is well recognised^8^ (Box 2). Genomic medicine and its contribution to precision medicine presents a unique set of challenges to a health system trying to keep up with the fast pace of advances over the past decade. An average of 17 years is required to integrate evidence‐based practices into routine health care, and genomics has exploded from widespread sequencing availability to TGA‐approved therapies requiring a precise genetic diagnosis in less than a decade.^20^ However, gaps in evidence, adoption, equity, and models of care remain, which have an impact on quality of care, cost effectiveness and resource utilisation^12^ (Box 1).

Box 2 **Using implementation science to plan translational genomics research**

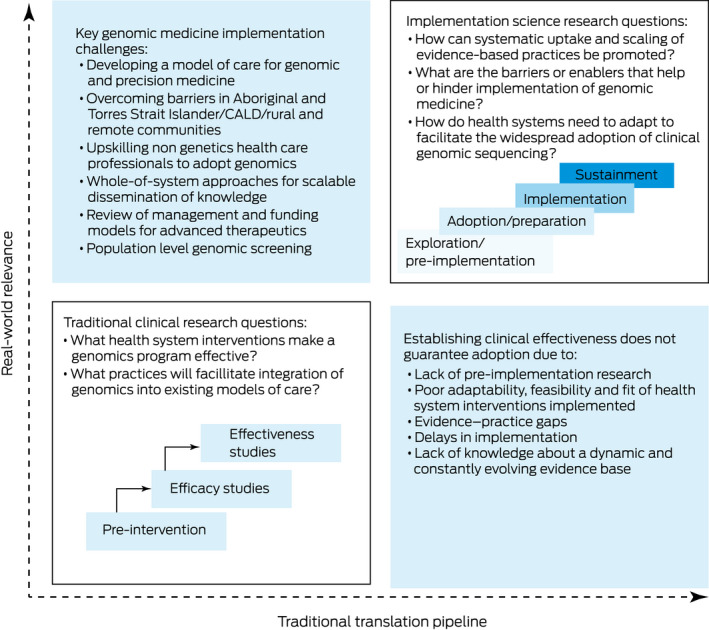

CALD = culturally and linguistically diverse. Figure adapted with permission from O'Connell et al.^19^ It highlights the key implementation challenges in genomic and precision medicine. It also illustrates the key differences between traditional clinical research, with its focus on intervention efficacy and effectiveness, and implementation science based research, which is focused on the “how” questions such as feasibility, sustainability and health system readiness for clinical adoption. These key differences help to address the problem that establishing clinical effectiveness alone does not guarantee clinical adoption due to many factors, such as evidence–practice gaps and localised barriers and needs.

## Genomic implementation: challenges ahead for Australia's precision medicine program

In a 2021 report^1^ on the new frontier of health in Australia, nationwide access to genomic testing and genomic counselling for all patients was recommended, but significant implementation barriers such as lack of genetics workforce were not addressed. Regulatory authority recommendations were made for improvement of availability of precision therapies, but key challenges in the adoption of genomic and precision medicine must be addressed to make these recommendations a reality (Box 2).

First, an effective, adaptable and sustainable model of clinical care for genomic and precision medicine is needed to address the limitations of the current genetics workforce. Second, the paucity of evidence about how best to address barriers to accessing genomic testing in Aboriginal and Torres Strait Islander people,^21^ culturally and linguistically diverse groups, and rural and remote communities must be considered, or we risk widening existing health care inequities and gaps in Australia. Third, upskilling non‐genetics professionals in genetics is urgently needed to enable mainstreaming of genomic medicine. Fourth, an investment in whole‐of‐system approaches, such as the Learning Healthcare Systems,^22^ is needed to facilitate wide‐scale education and knowledge translation at a local level. Fifth, a review of existing management and funding models for often costly advanced therapeutics is needed, with consideration for the whole patient journey, including the required genomic diagnostics and care before a patient is eligible for therapy. Finally, the introduction of genomic medicine into primary care and population screening will challenge existing health care infrastructure. Health care professions and the public need to be well equipped to understand genomics and engage in debate about ethical issues that shape our society.

## A call to action: implementation science research in precision medicine

The challenges to implementing genomic and precision medicine in Australia provide an opportunity for translational research informing policy and practice. The relatively new discipline of implementation science is defined as “the scientific study of methods to promote the systematic uptake of research findings and other evidence‐based practices into routine practice, and, hence, to improve the quality and effectiveness of health services”.^23^ It aims to gain generalisable knowledge in a health system that could be widely applied to different providers, clinics or health systems.^24^ Implementation research can deal with complex health services issues more effectively than traditional clinical effectiveness research (Box 2).^9^

Implementation science uses theories (explaining mechanisms), models (descriptive processes) and frameworks (organisational structures and relationships)^25^ to plan research through stages of exploration or pre‐implementation, adoption, implementation and sustainability (Box 2).^19^ It focuses on identifying the barriers and facilitators (or determinants) to target and change, matching implementation strategies to these determinants, and testing strategies in real‐world settings (Box 1 and Box 2),^25^ as outlined in models such as the knowledge‐to‐action cycle.^4^ Implementation outcomes such as acceptability, adoption, sustainment and scalability are measured at individual provider, health service, and system levels.^7^ This structured approach can identify the mechanisms of behaviour change by selecting relevant strategies to improve evidence‐based practice adoption and adapting these for new contexts beyond the initial setting.^26^

An implementation science approach can address many of the already identified barriers and gaps in precision medicine (Box 1 and Box 2). Yet studies that explore these systemic adoption issues are a minority of funded genomics research. A review of genomic grants funded by the National Institutes of Health found that only 1.75% were implementation science studies.^27^ The Australian Genomics project^28^ seeks to translate genomic research into clinical practice, and is using implementation approaches and selected flagship models^29^ to investigate the uptake of genomics.^30^ The studies have identified critical factors such as a learning health care systems approach to audit and feedback, collaboration through networks, and leadership and culture in delivering genomic health care.

Other Australian research groups have demonstrated the outstanding success of genomic care in new genetic diagnoses and management pathways.^31^, ^32^, ^33^ This has led to direct implementation of genomic diagnostic testing,^34^ supported by state‐based funding, and further prompted strategic implementation projects; for example, the NSW Health Genomics Strategy,^35^ which facilitated the first TGA‐approved clinical *in vivo* gene therapy in Australia for retinal dystrophy^36^ and gene therapy for spinal muscular atrophy in newborns.

Another Australian example is the national implementation science evaluation of a mainstreaming initiative to integrate routine genetic testing for breast and ovarian cancer.^37^ The barriers identified can be generalised to other areas of genomic medicine, including the practitioner (role delineation) and health care system (funding and infrastructure) levels (Box 1), and identifying strategies to overcome barriers such as the use of genomic “champions”, electronic tracking systems, and defined care pathways.^37^

A systematic review^3^ of global health system interventions to embed genomic medicine into oncology identified that new models of care, interdisciplinary collaborations, and adaptable learning health systems are needed. Undertaking pre‐implementation research, which includes engagement with stakeholders, codesigning strategies, and assessment of readiness for change within organisations and the local context (or setting),^38^ would allow for health care planning and service delivery approaches that support and sustain equitable genomic testing adoption.^3^ This could make a significant difference, for example, in paediatrician‐ordered genomic sequencing in children with intellectual disability. Despite funding for paediatric genomic testing being available since 2020 and tailored educational materials (ie, implementation strategies), there has been a slow and patchy uptake.^39^, ^40^ Barriers identified include a lack of time for informed genomic consent and completion of paperwork by paediatricians, which is not addressed in the funding model (Box 1).

To ensure effective models of genomic care are created, there is an urgent need for local hospital and health service and state‐based genomic medicine implementation research (Box 1). Such research would allow evidence generation for optimal adoption, knowledge of factors affecting practice, and would inform policy about precision medicine program design. A focus on pre‐implementation research commensurate with the introduction of new Medicare numbers for genomics will help define the best scalable models of care to implement genomics into routine practice. This call to action will bring the benefits of precision medicine for all Australians.

## Open access

Open access publishing facilitated by The University of Sydney, as part of the Wiley – The University of Sydney agreement via the Council of Australian University Librarians.

## Competing interests

No relevant disclosures.

## Provenance

Not commissioned; externally peer reviewed.
